# Impact of components of metabolic syndrome on the risk of adverse renal outcomes in patients with atrial fibrillation: a nationwide cohort study

**DOI:** 10.3389/fcvm.2023.1208979

**Published:** 2023-10-17

**Authors:** Soonil Kwon, So-Ryoung Lee, Eue-Keun Choi, Seung-Woo Lee, Jin-Hyung Jung, Kyung-Do Han, Hyo-Jeong Ahn, Seil Oh, Gregory Y. H. Lip

**Affiliations:** ^1^ Department of Internal Medicine, Seoul National University Hospital, Seoul, Republic of Korea; ^2^ Department of Internal Medicine, Seoul National University College of Medicine, Seoul, Republic of Korea; ^3^ Department of Medical Statistics, College of Medicine, The Catholic University of Korea, Seoul, Republic of Korea; ^4^ Department of Statistics and Actuarial Science, Soongsil University, Seoul, Republic of Korea; ^5^ Liverpool Centre for Cardiovascular Science at University of Liverpool, Liverpool John Moores University and Liverpool Chest & Heart Hospital, Liverpool, United Kingdom; ^6^ Department of Clinical Medicine, Aalborg University, Aalborg, Denmark

**Keywords:** atrial fibrillation, end-stage renal disease, epidemiology, metabolic syndrome, risk factor

## Abstract

**Background:**

The renal effect of metabolic syndrome components is unclear in patients with atrial fibrillation. This study aimed to investigate the association between metabolic syndrome components and incident end-stage renal disease among patients with atrial fibrillation.

**Methods:**

A total of 202,434 atrial fibrillation patients without prevalent end-stage renal disease were identified from the National Health Insurance Service database between 2009 and 2016. We defined the metabolic score range from 0 to 5 points such that a patient received every 1 point if the patient met each component listed in the diagnostic criteria of metabolic syndrome. The population was divided into 6 groups: MS_0_–MS_5_ for a metabolic score of 0–5, respectively. Multivariate Cox regression analysis was used to estimate the risks of end-stage renal disease.

**Results:**

There were 12,747, 31,059, 40,361, 48,068, 46,630, and 23,569 patients for MS_0_–MS_5_, respectively. Compared with MS_0_, MS_5_ had a higher CHA_2_DS_2_-VASc score (3.8 vs. 1.0) (*P* < .001). During a median follow-up of 3.5 years, compared with MS_0_, MS_1_–MS_5_ were associated with a gradually increasing incidence of end-stage renal disease, in relation to an increase in the metabolic score, (log-rank *P* < .001). After multivariate adjustment, a higher metabolic score was associated with a greater risk of incident end-stage renal disease: adjusted hazard ratio [95% confidence interval] = 1.60 [0.78–3.48], 2.08 [1.01–4.31], 2.94 [1.43–6.06], 3.71 [1.80–7.66], and 4.82 [2.29–10.15], for MS_1_–MS_5_, respectively.

**Conclusions:**

Metabolic syndrome components additively impacts the risk of incident end-stage renal disease among patients with atrial fibrillation.

## 1. Introduction

Atrial fibrillation (AF) and chronic kidney disease (CKD) have common risk factors, and they impact the progression of each other (1). AF is associated with an increased risk of CKD (2), while AF concurrent with CKD accelerates renal function decline, which may lead to renal failure (3). Renal failure has a crucial impact on AF management by limiting the choice of antiarrhythmic agents and oral anticoagulants that are used for stroke prevention (4). Relative to normal renal function, end-stage renal disease (ESRD) increases the risk of stroke or hemorrhage in patients with AF by 1.8-fold (5). Therefore, predicting a high-risk population for incident ESRD is important for managing AF.

Metabolic disorders are the leading cause of ESRD (6). In particular, hypertension and diabetes mellitus are common comorbidities in patients with AF, with prevalence rates as high as 68% and 23%, respectively (7). Furthermore, metabolic syndrome is prevalent in up to 22.7% of the AF population (8). However, the evidence for an association between metabolic syndrome and incident ESRD in patients with AF is scarce. Metabolic syndrome is a comprehensive disorder that includes obesity, lipid imbalance, hypertension, and impaired glycemic control (9). Although some studies have reported that hypertension or diabetes mellitus increases the risk of incident ESRD (10), there remains a lack of evidence on whether different types of metabolic disorders contribute additively to an increased risk of ESRD in patients with AF.

Considering that most patients with AF have multiple comorbidities, incident ESRD may be predicted better by stratifying patients according to the severity of metabolic disorders. The definition of metabolic syndrome includes five components: increased waist circumference, elevated triglycerides, low high-density lipoprotein cholesterol, increased blood pressure, and impaired fasting blood glucose (9). In this context, the status of metabolic syndrome may be considered severer when more criteria are met. Investigating the impact of each criterion on incident ESRD may help identify patients with AF who are at a high risk of ESRD.

This study aimed to investigate the impact of metabolic syndrome components on the risk of incident ESRD in patients with AF using a nationwide cohort study.

## 2. Materials and methods

This retrospective cohort study used the health checkup data from 2009 to 2016 available at the National Health Insurance Service (NHIS) of the Republic of Korea. Korean adults aged ≥40 years are subject to routine health checkups biannually. These health checkups are supported by the NHIS, which is the single public health insurer in Korea. The health check-up database comprises demographic information, history of claimed diagnostic codes, results of simple blood tests, and surveys on health habits. The use of the NHIS database for cardiovascular research has been described elsewhere previously (11). This study conformed to the Declaration of Helsinki revised in 2013, and was approved by the Institutional Review Board of the Seoul National University Hospital (No. 2301–030–1392). The requirement for informed consent was waived because of the nature of the study (anonymized data used retrospectively).

### 2.1. Study population

**Figure 1:**
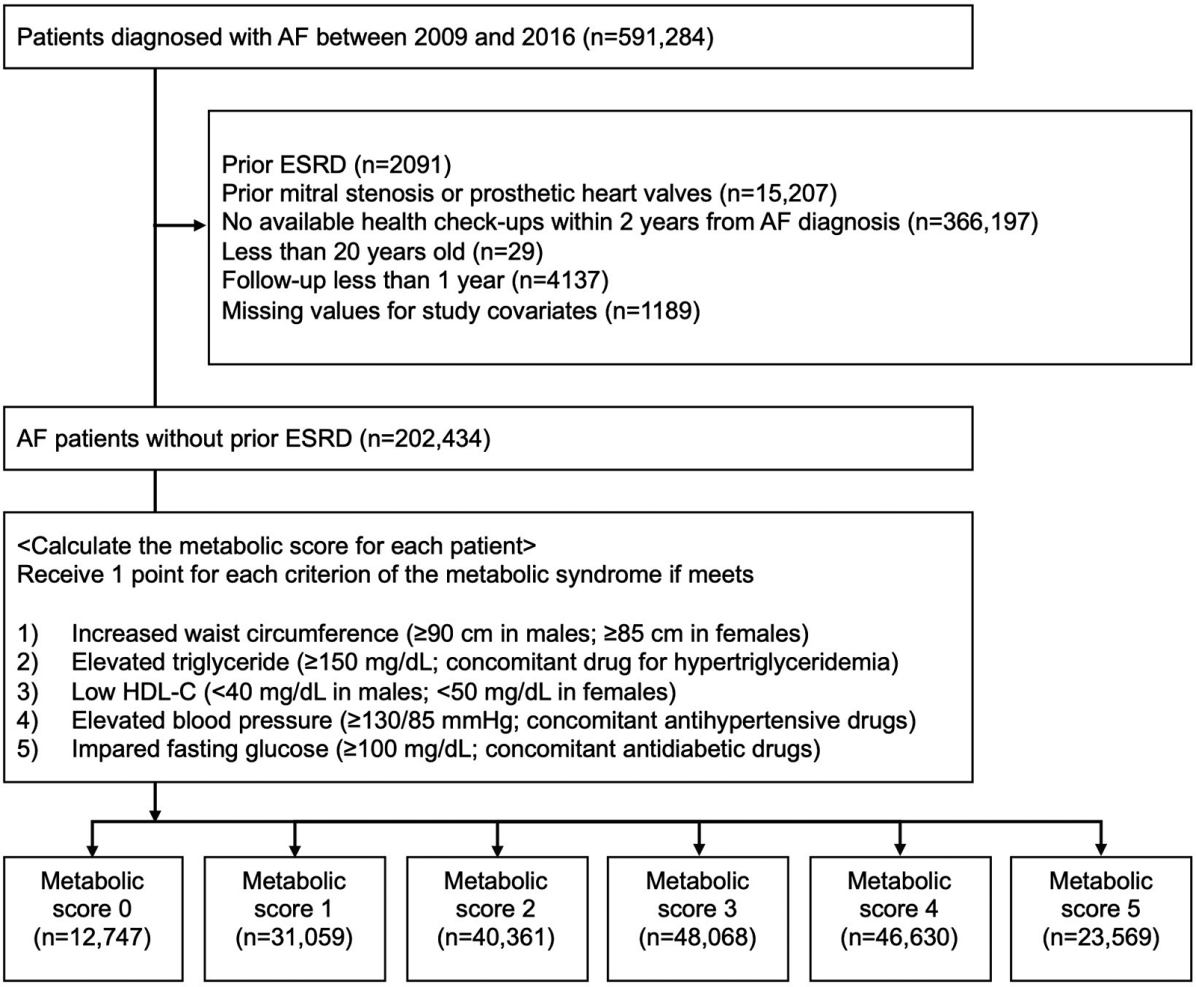
The flowchart of the study population. The study population was divided into 6 groups (MS0–MS5) according to each metabolic score (0–5). AF, atrial fibrillation; ESRD, end-stage renal disease; HDL-C, high-density lipoprotein cholesterol.

The flowchart of the study population is shown in Figure 1. From the database, we extracted the data of patients diagnosed with AF during 2009 to 2016. We excluded the following populations: (1) patients with prevalent ESRD (*n* = 2091); (2) patients with mitral stenosis or prosthetic heart valves (*n* = 15,207); (3) patients who had no available health checkups within 2 years from the diagnosis of AF (*n* = 366,197); (4) patients aged <20 years (*n* = 29); (5) patients with missing values for study covariates (*n* = 1189); and (6) patients with a follow-up period <1 year (*n* = 4137). Consequently, 202,434 patients with AF without prior ESRD were investigated.

### 2.2. Definitions of metabolic syndrome and the metabolic score

We defined the metabolic score range from 0 to 5 points, such that a patient received 1 point if he/she met each diagnostic criteria for metabolic syndrome. The diagnostic criteria of metabolic syndrome were defined based on an international guideline (9), with the adoption of the criteria for increased waist circumference according to the Korean Society for the Study of Obesity (12). The five diagnostic criteria are summarized in Table 1. The study population was then categorized into six groups (MS_0_ to MS_5_) according to their metabolic scores (0–5).

**Table 1:** The definition of diagnostic criteria for metabolic syndrome (9).

Measure	Categorical cut points
Increased waist circumference	≥90 cm in males; ≥85% cm in females (12)
Elevated triglycerides	≥150 mg/dl (1.7 mmol/L); concomitant use of drugs for hypertriglyceridemia
Low HDL cholesterol	<40 mg/dl (1.0 mmol/L) in males; <50 mg/dl (1.3 mmol/L) in females
Elevated blood pressure	Systolic ≥130 mmHg and/or diastolic ≥85 mmHg; concomitant use of antihypertensive drugs
Impaired fasting blood glucose	Fasting plasma glucose ≥100 mg/dl (5.6 mmol/L); concomitant use of antidiabetic drugs

HDL, high-density lipoprotein.

### 2.3. Study covariates

The study covariates were measured using data from the NHIS database. Individual covariates were obtained at the index health checkup, and Supplementary Table S1 summarizes their detailed definitions. General information regarding the population's characteristics, including age, sex, height, body weight, CHA_2_DS_2_-VASc scores, alcohol consumption (yes/no), smoking (yes/no), regular exercise (yes/no), and low-income status (yes/no) was collected. Comorbidities were investigated using established diagnostic codes, including diabetes mellitus, ischemic heart disease, heart failure, ischemic stroke, peripheral artery disease, dyslipidemia, diabetes mellitus, chronic obstructive pulmonary disease, CKD, and any malignancy. Diagnostic codes were encoded according to the International Classification of Diseases, Tenth Revision, Clinical Modification. Data about concomitant medication, including oral anticoagulants (warfarin or direct oral anticoagulants), antiplatelet agents (aspirin or P_2_Y_12_ inhibitors), antidiabetic drugs (sulfonylurea, meglitinide, metformin, thiazolidinedione, alpha-glucosidase inhibitors, dipeptidyl peptidase−4 inhibitors, and insulin), antihypertensive drugs (angiotensin receptor blockers, angiotensin-converting enzyme inhibitors, beta-blockers, calcium channel blockers, and diuretics), and statins were obtained from the claims database. Data for blood pressure, fasting blood glucose, total cholesterol, high- and low-density lipoprotein cholesterol (HDL-C, LDL-C), triglyceride, serum creatinine, and estimated glomerular filtration rate (eGFR) were obtained from the health checkup database.

### 2.4. Study outcomes and the follow-up

The primary outcome was incident ESRD, which was defined as having a diagnostic code (N18.5 or Z49) with hemodialysis or peritoneal dialysis ≥2 times during the follow-up period. Individuals were right-censored when the primary outcome occurred and were followed up from the index health checkup to December 31st, 2018.

### 2.5. Statistical analyses

Baseline characteristics were compared across the six groups (MS_0_–MS_5_) using a one-way analysis of variance or Kruskal–Wallis *H* test according to the type of covariate. Survival analysis was performed using the Kaplan–Meier method, and a log-rank test was used to compare survival across the six groups. Crude incidence rates (IRs) of ESRD were calculated in 1000 person-years. The risk of incident ESRD was estimated by multivariate Cox regression analyses and reported as adjusted hazard ratios (HRs) with 95% confidence intervals (CIs). The final model used covariates, including age, sex, body mass index, low-income status, health habits (including alcohol consumption, smoking, and regular exercise), comorbidities (including ischemic heart disease, heart failure, stroke, peripheral artery disease, chronic obstructive pulmonary disease, and any malignancy), concomitant drug use (including oral anticoagulants and antiplatelet agents), the five metrics used in the definition of metabolic syndrome (including waist circumference, fasting blood glucose, blood pressure, triglyceride, and HDL-C), and renal function (eGFR). Subgroup analyses were performed for sex, strata of CHA_2_DS_2_-VASc scores (0–1 vs. ≥2), and strata of eGFR (≥60 vs. <60 ml/kg/1.73 m^2^) and concurrent use of oral anticoagulant (OAC) (any vs. none). All statistical analyses were performed using SAS version 9.3 (SAS Institute, Cary, NC, USA). Two-sided *P* < .05 were used to reject the null hypothesis.

### 2.6. Additional analyses

To investigate the impact of each diagnostic criterion of metabolic syndrome on incident ESRD, the study population was divided according to the presence or absence of each diagnostic criterion of metabolic syndrome. The risk of incident ESRD was compared across the five groups based on patients meeting each diagnostic criterion. The impact of systolic blood pressure and fasting blood glucose levels on the incident ESRD risk was visualized using cubic spline curves.

We also calculated the area under the receiver operating characteristics curves (AUROCs) of metabolic scores and five components of metabolic syndrome (waist circumference, fasting blood glucose, systolic blood pressure, HDL-C, and triglyceride) to predict incident ESRD at 1-year.

### 2.7. Sensitivity analyses

A total of 11 statistical models were created using different sets of covariates for model adjustment, and their results were compared with those of the final model. A complete list of the statistical models is presented in Supplementary Table S2. We also compared the results of the final model with those of the other three models each with different covariates for renal function: eGFR, presence of CKD diagnosis (N18), and presence of decreased eGFR (<60 ml/kg/1.73 m^2^) for Model 8, 9, and 10, respectively.

## 3. Results

### 3.1. Baseline characteristics

In total, 202,434 patients with AF without prior ESRD were included in the analysis. The study population was divided into six groups (MS_0_–MS_5_) according to their metabolic scores (0–5), with *n* = 12,747 (6.3%), 31,059 (15.3%), 40,361 (19.9%), 48,068 (23.7%), 46,630 (23.0%), and 23,569 (11.6%) in each subgroup, respectively. The population's mean age, male proportion, and CHA_2_DS_2_-VASc score were 63.5 ± 12.1 years, 49.5%, and 2.8 ± 1.6, respectively.

As the metabolic score increased, the population's age, body mass index, and comorbidities (except malignancy) also increased (Table 2); mean ages increased from 52.5 years (MS_0_) to 66.3 years (MS_5_); body mass index from 22.0 kg/m^2^ (MS_0_) to 27.7 kg/m^2^ (MS_5_); all *P* < .001. Furthermore, the concomitant medication (oral anticoagulants, antiplatelet agents, antidiabetic drugs, antihypertensive drugs, and statins) also increased in relation to metabolic score (from MS_0_ to MS_5_, Table 2); all *P* < .001. Among the laboratory test results, blood pressure, fasting blood glucose, triglyceride, and serum creatinine increased as the metabolic score increased, while total cholesterol, HDL-C, LDL-C, and eGFR decreased (Table 2); all *P* < .001.

**Table 2:** Baseline characteristics of the study population according to the metabolic score.

	MS_0_ (*n* = 12,747)	MS_1_ (*n* = 31,059)	MS_2_ (*n* = 40,361)	MS_3_ (*n* = 48,068)	MS_4_ (*n* = 46,630)	MS_5_ (*n* = 23,569)	*P*
Demographics							
Age, year	52.5 ± 14.8	60.7 ± 14.0	63.3 ± 12.7	64.7 ± 11.8	66.0 ± 10.9	66.3 ± 10.4	<.001
Men, %	6,808 (53.4)	19,301 (62.1)	25,103 (62.2)	28,403 (59.1)	27,150 (58.2)	13,168 (55.9)	<.001
Height, cm	163.8 ± 8.9	162.9 ± 9.4	162.5 ± 9.6	161.8 ± 9.7	161.6 ± 9.7	162.0 ± 9.8	<.001
Weight, kg	59.2 ± 9.5	60.8 ± 10.3	63.3 ± 11.4	64.5 ± 11.9	66.2 ± 11.9	72.9 ± 11.8	<.001
Body mass index, kg/m^2^	22.0 ± 2.5	22.8 ± 2.7	23.9 ± 3.0	24.5 ± 3.2	25.3 ± 3.2	27.7 ± 3.1	<.001
CHA_2_DS_2_-VASc score							<.001
Mean	1.0 ± 1.0	2.1 ± 1.5	2.5 ± 1.7	3.0 ± 1.7	3.5 ± 1.7	3.8 ± 1.7	<.001
Median	1 (0–1)	2 (1–3)	2 (1–4)	3 (2–4)	3 (2–5)	4 (3–5)	<.001
Current smoker, %	2,026 (15.9)	4,797 (15.4)	5,974 (14.8)	6,645 (13.8)	6,296 (13.5)	2,953 (12.5)	<.001
Alcohol drinker, %	4,647 (36.5)	10,780 (34.7)	13,925 (34.5)	14,977 (31.2)	13,727 (29.4)	6,977 (29.6)	<.001
Regular exercise, %	2,807 (22.0)	6,763 (21.8)	8,468 (21.0)	9,862 (20.5)	9,519 (20.4)	4,521 (19.2)	<.001
Low-income status, %	2,482 (19.5)	6,300 (20.3)	8,336 (20.7)	9,932 (20.7)	9,736 (20.9)	5,128 (21.8)	<.001
Comorbidities, %							
Hypertension	0 (0)	16,981 (54.7)	26,904 (66.7)	38,031 (79.1)	42,184 (90.5)	22,656 (96.1)	<.001
Ischemic heart disease	187 (1.5)	689 (2.2)	1,081 (2.7)	2,254 (4.7)	3,101 (6.7)	1,569 (6.7)	<.001
Heart failure	1,031 (8.1)	5,501 (17.7)	8,339 (20.7)	11,281 (23.5)	12,325 (26.4)	6,589 (28.0)	<.001
Ischemic stroke	518 (4.1)	2,255 (7.3)	4,058 (10.1)	7,398 (15.4)	8,781 (18.8)	4,414 (18.7)	<.001
Peripheral artery disease	1,016 (8.0)	4,564 (14.7)	7,093 (17.6)	10,055 (20.9)	11,442 (24.5)	6,216 (26.4)	<.001
Dyslipidemia	871 (6.8)	2,214 (7.1)	6,858 (17.0)	24,260 (50.5)	35,137 (75.4)	20,623 (87.5)	<.001
Diabetes mellitus	0 (0)	1,163 (3.7)	5,799 (14.4)	8,508 (17.7)	17,448 (37.4)	13,424 (57.0)	<.001
COPD	1,568 (12.3)	5,268 (17.0)	7,151 (17.7)	8,572 (17.8)	8,848 (19.0)	4,552 (19.3)	<.001
Chronic kidney disease	600 (4.7)	3,042 (9.8)	5,310 (13.2)	7,850 (16.3)	9,545 (20.5)	5,557 (23.6)	<.001
Any malignancy	943 (7.4)	1,933 (6.2)	2,302 (5.7)	2,332 (4.9)	2,116 (4.5)	1,018 (4.3)	<.001
Concomitant drug, %							
Oral anticoagulant	1,530 (12.0)	6,341 (20.4)	9,412 (23.3)	13,563 (28.2)	14,870 (31.9)	8,117 (34.4)	<.001
Warfarin	1,313 (10.3)	5,140 (16.6)	7,397 (18.3)	10,399 (21.6)	10,962 (23.5)	5,727 (24.3)	<.001
DOAC	268 (2.1)	1,563 (5.0)	2,681 (6.6)	4,229 (8.8)	5,181 (11.1)	3,159 (13.4)	<.001
Antiplatelet agent	4,088 (32.1)	16,008 (51.5)	22,968 (56.9)	31,443 (65.4)	33,103 (71.0)	17,203 (73.0)	<.001
Aspirin	3,852 (30.2)	14,929 (48.1)	21,257 (52.7)	28,698 (59.7)	30,071 (64.5)	15,566 (66.0)	<.001
P_2_Y_12_ inhibitors	895 (7.0)	3,893 (12.5)	6,247 (15.5)	12,051 (25.1)	14,742 (31.6)	7,830 (33.2)	<.001
Antidiabetic drugs	0 (0)	822 (2.7)	4,304 (10.7)	6,551 (13.6)	14,694 (31.5)	11,520 (48.9)	<.001
Antihypertensive drugs	0 (0)	15,657 (50.4)	24,951 (61.8)	36,020 (74.9)	40,670 (87.2)	22,178 (94.1)	<.001
Statin	0 (0)	0 (0)	3,540 (8.8)	21,341 (44.4)	33,558 (72.0)	20,259 (86.0)	<.001
Laboratory tests							
SBP, mmHg	112.3 ± 9.5	122.5 ± 14.7	125.6 ± 15.4	126.8 ± 15.6	128.6 ± 15.5	130.6 ± 15.7	<.001
DBP, mmHg	70.3 ± 7.4	75.8 ± 10.0	77.3 ± 10.3	77.8 ± 10.4	78.2 ± 10.5	78.9 ± 10.5	<.001
Fasting blood glucose, mg/dl	88.2 ± 7.3	93.1 ± 15.3	101.7 ± 23.6	102.8 ± 25.4	113.4 ± 32.7	124.1 ± 35.5	<.001
Total cholesterol, mg/dl	188.8 ± 32.9	188.3 ± 34.3	189.4 ± 39.3	182.1 ± 43.5	174.9 ± 44.4	171.1 ± 41.7	<.001
HDL-C, mg/dl	60.9 ± 16.2	57.4 ± 15.8	53.7 ± 17.3	50.7 ± 17.8	49.0 ± 17.2	48.1 ± 30.2	<.001
LDL-C, mg/dl	111.6 ± 34.6	112.5 ± 77.5	112.6 ± 42.6	103.9 ± 45.2	95.7 ± 44.2	91.0 ± 39.5	<.001
Triglyceride, mg/dl	82.9 ± 28.8	96.9 ± 50.6	120.2 ± 74.9	144.0 ± 98.5	158.8 ± 107.7	168.6 ± 112.1	<.001
Serum creatinine, mg/dl	0.91 ± 0.65	0.95 ± 0.63	0.98 ± 0.64	0.99 ± 0.80	1.01 ± 0.77	1.02 ± 0.53	<.001
Estimated GFR, ml/min/1.73m^2^	89.6 ± 28.3	84.7 ± 29.8	82.1 ± 28.3	79.9 ± 31.2	77.3 ± 29.7	75.6 ± 27.4	<.001

COPD, chronic obstructive pulmonary disease; DBP, diastolic blood pressure; DOAC, direct oral anticoagulant; GFR, glomerular filtration rate; HDL-C, high-density lipoprotein cholesterol; LDL-C, low-density lipoprotein cholesterol; SBP, systolic blood pressure.

MS_0_–MS_5_ denotes the populations with a metabolic score of 0–5, accordingly.

Data are presented as *n* (%), mean ± standard deviation, or median (interquartile range). *P*-values are for across six groups.

### 3.2. Impact of the metabolic score on the risk of incident ESRD among AF patients

**Figure 2:**
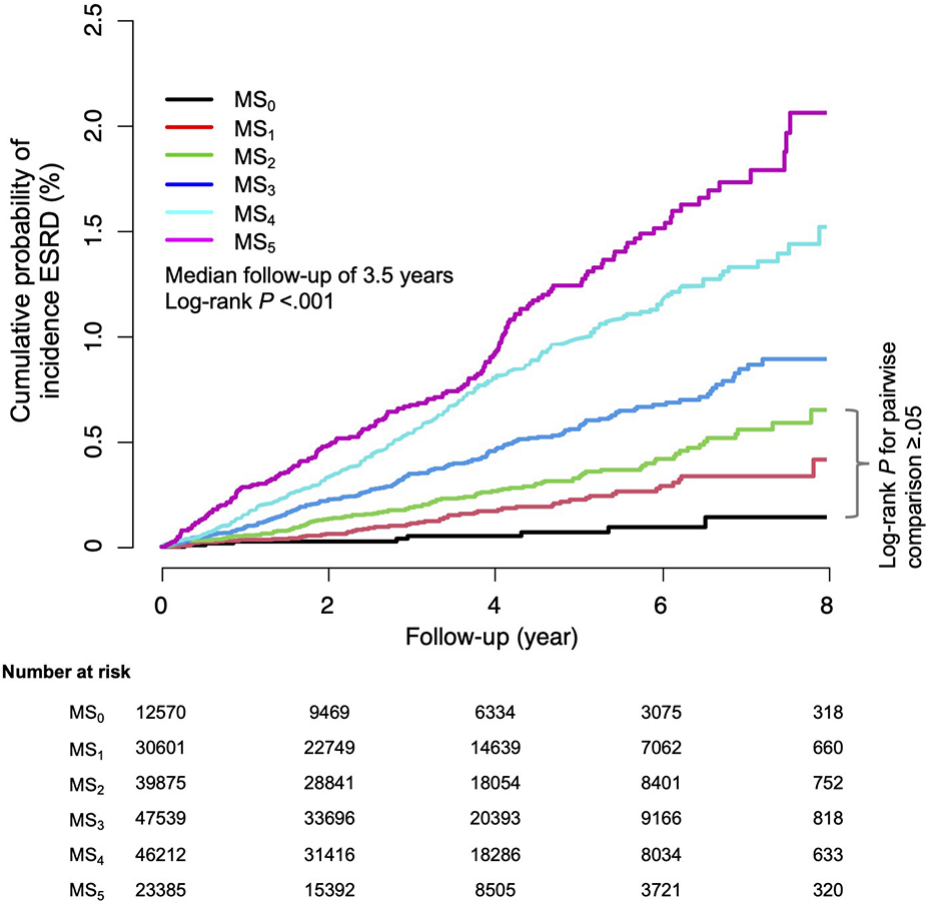
The cumulative incidence of ESRD among AF patients according to the metabolic score. The crude incidence rates of ESRD increased significantly as metabolic score increased (*P*-for-trend <.001). AF, atrial fibrillation; ESRD, end-stage renal disease; MS, metabolic score.

During a median follow-up of 3.5 (interquartile ranges, 1.7–5.6) years, the crude incidence rate of ESRD among AF patients gradually increased for higher metabolic scores; 0.16, 0.45, 0.70, 1.13, 1.87, and 2.48 per 1000 person-years for MS_0_–MS_5_, respectively. There was a significant difference in ESRD-free survival across the five groups (log-rank *P* < .001), although there was a comparable result between MS_0_, MS_1_, and MS_2_ (pairwise log-rank *P* ≥ 0.05) (Figure 2).

**Figure 3:**
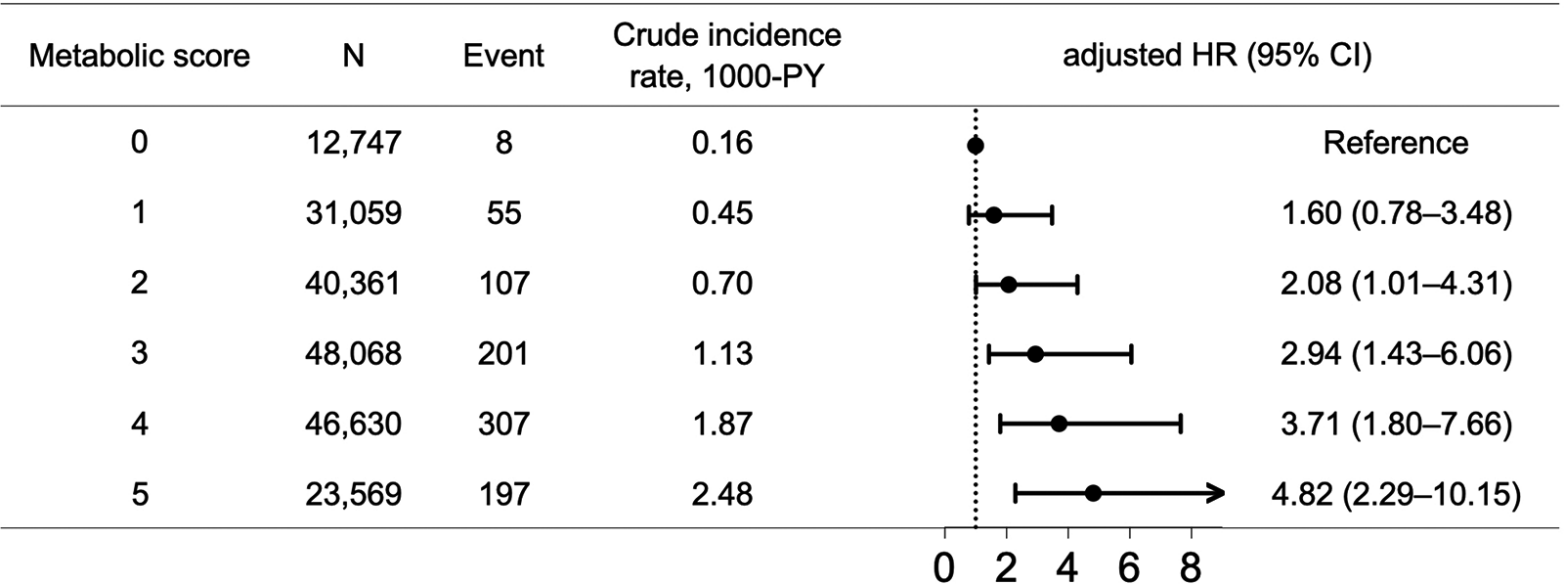
The risks of incident ESRD among AF patients across metabolic scores. There was a trend of increasing risks of incident ESRD for higher metabolic scores (*P*-for-trend <.001). AF, atrial fibrillation; CI, confidence interval; ESRD, end-stage renal disease; HR, hazard ratio; PY, person-year.

Metabolic syndrome was associated with a 2.9-fold increase in the risk of ESRD [adjusted HR 2.94 (95% CI, 1.43–6.06)]. After multivariate adjustment, the final model showed a trend for higher risk of incident ESRD in relation to higher metabolic scores (*P*-for-trend <.001) (Figure 3). Compared to MS_0_, all others (except MS_1_) were associated with significantly increased risks of incident ESRD (adjusted HR, 1.60 [95% CI 0.78–3.48], 2.08 [1.01–4.31], 2.94 [1.43–6.06], 3.71 [1.80–7.66], and 4.82 [2.29–10.15] for MS_1_–MS_5_, respectively (Figure 3).

### 3.3. Impact of metabolic syndrome component on incident ESRD

**Figure 4:**
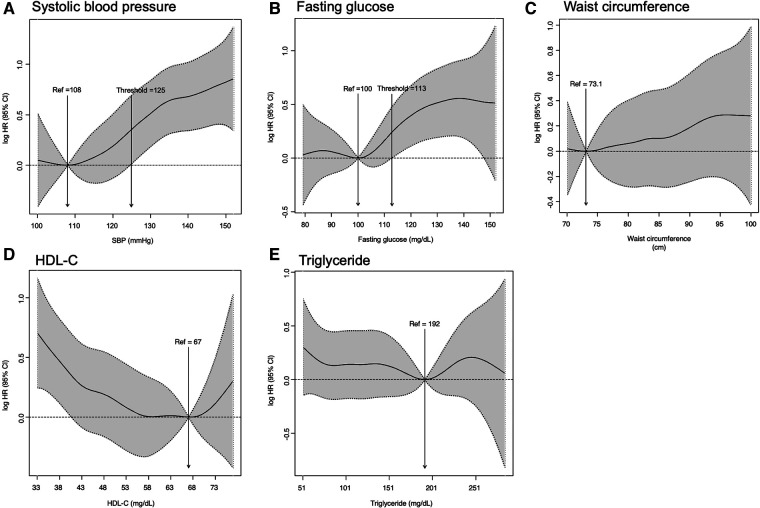
Impact of metabolic components on the risks of incident ESRD; (A) systolic blood pressure; (B) fasting blood glucose; (C) waist circumference; (D) HDL-C; (E) triglyceride. CI, confidence interval; ESRD, end stage renal disease; HDL-C, high-density lipoprotein cholesterol; HR, adjusted hazard ratio; Ref, reference; SBP, systolic blood pressure.

Among the five metabolic syndrome components, the increased risk of ESRD due to metabolic syndrome was primarily driven by elevated blood pressure; adjusted HRs (95% CI) in decreasing order, 2.20 (1.60–3.03), 1.66 (1.42–1.95), 1.61 (1.36–1.91), and 1.19 (1.02–1.40) for elevated blood pressure, impaired fasting blood glucose, low HDL-C, and elevated triglycerides, respectively (Supplementary Figure S1). In contrast, increased waist circumference did not significantly impact the risk of ESRD [adjusted HR 1.12 (95% CI, 0.90–1.38)]. The cubic spline curves showed that the systolic blood pressure and fasting blood glucose thresholds for increased ESRD risks were 125 mmHg and 113 mg/dl, respectively (Figure 4).

### 3.4. Subgroup analyses

There was no significant interaction for strata of CHA_2_DS_2_-VASc scores (0–1 vs. ≥2) and sex (*P*-for-interaction = .966 and.838, respectively) (Supplementary Table S3). Compared to the subgroup with preserved eGFR (≥60 ml/kg/1.73 m^2^), the trend of increased risk for ESRD in relation to higher metabolic scores was accentuated in the subgroup with decreased eGFR (<60 ml/kg/1.73 m^2^) (Supplementary Table S3). For the subgroup without OAC use, there was a trend of increasing risks of incident ESRD with higher metabolic scores (Supplementary Table S3). Conversely, for the subgroup with OAC use, no definitive trend was observed. However, there was no significant interaction (*P*-for-interaction = .115).

### 3.5. Sensitivity analyses

The main result was compared with different multivariate Cox regression analyses (Models 1–10). Regardless of the statistical models, there was a consistent trend of increasing risk of ESRD in relation to higher metabolic scores. However, the magnitudes of HRs decreased as more covariates were adjusted for (all *P*-for-trend < .001) (Supplementary Table S4). Consistent results were observed across the models regardless of the covariates representing renal function: eGFR, the presence of CKD diagnosis, or the presence of decreased eGFR (<60 ml/kg/1.73 m^2^) for models 8, 9, and 10, respectively (Supplementary Table S4).

### 3.6. Performance of metabolic scores to predict incident ESRD at 1-year

The AUROCs of metabolic scores and the five components of metabolic syndrome are presented in Table 3. Compared to the metabolic components, except for systolic blood pressure, metabolic scores showed a significantly higher AUROC (0.68 with a 95% CI of 0.65–0.72). Compared to the AUROC of systolic blood pressure, that of metabolic scores showed a higher value with marginal significance (AUROC = 0.68 [95% CI 0.65–0.72] vs. 0.64 [95% CI 0.61–0.68], *P* = .096).

**Table 3:** Comparison of AUROCs of metabolic scores and components of metabolic syndrome to predict incident ESRD at 1-year.

	AUROC (95% CI)	*P*
Metabolic scores	0.68 (0.65–0.72)	Reference
Systolic blood pressure	0.64 (0.61–0.68)	.096
HDL-C	0.62 (0.59–0.66)	.020
Fasting blood glucose	0.59 (0.54–0.64)	<.001
Waist circumference	0.57 (0.53–0.61)	<.001
Triglyceride	0.53 (0.49–0.57)	<.001

AUROC, the area under the receiver operating characteristics curve; CI, confidence interval; HDL-C, high-density lipoprotein cholesterol.

## 4. Discussion

This study investigated the impact of metabolic syndrome on the risk of incident ESRD in patients with AF using a nationwide cohort. Our principal findings were: (1) metabolic syndrome was associated with a 2.9-fold increase in the risk for ESRD; (2) there was a trend of increasing risks of incident ESRD as metabolic scores increased; and (3) the increased risk of ESRD due to metabolic syndrome was mainly driven by elevated blood pressure and impaired fasting blood glucose. To our knowledge, this is the first study to demonstrate an association between metabolic syndrome and incident ESRD in a nationwide AF population.

AF and renal function are closely interrelated (1, 2). Recent retrospective cohort studies showed a bidirectional association between AF and renal function (3, 13). While renal dysfunction was associated with an increased risk of AF, it may further aggravate the underlying renal dysfunction (3, 13), especially when blood pressure is poorly controlled (10). As a result, AF is vulnerable to renal failure.

Appropriate medical management becomes difficult if renal failure coexists with AF. First, the medical management of rhythm control is limited. Flecainide or sotalol are not recommended because of their dependency on renal excretion (14). Second, renal failure limits the optimal drug choice for stroke prevention and rhythm control in patients with AF. Although warfarin is associated with a higher risk of bleeding compared to direct oral anticoagulants, it remains the mainstream treatment for stroke prevention in patients with AF and ESRD because direct oral anticoagulants are contraindicated due to their dependency on renal excretion (15). However, warfarin also accelerates calcific uremic arteriolopathy in ESRD, and increases mortality (16). While apixaban is approved by the Food and Drug Administration for stroke prevention among patients with AF requiring dialysis (17), the evidence is relatively weaker than its indicated general use among non-dialysis patients (18). Therefore, underlying renal failure complicates stroke prevention in patients with AF.

The medical management of AF with concurrent renal failure is a challenging task. Although there have been studies that reported the association between individual components of metabolic syndrome and ESRD, the impact of their interaction on ESRD is not well understood, especially in patients with AF. In the general population, some components of metabolic syndrome, such as hypertension and diabetes mellitus, are well-known risk factors for ESRD (19, 20). However, hypertension and diabetes often coexist with other metabolic disorders such as obesity and dyslipidemia. Therefore, a more comprehensive approach is necessary to improve the prediction of ESRD.

Metabolic syndrome, which is a broader concept (compared to hypertension or diabetes mellitus), has been reported to increase the risk of CKD by 34% in the Chinese population (21). In contrast, our study showed that metabolic syndrome increased the risk of ESRD by 2.9-fold. The higher impact of metabolic syndrome on ESRD could be due to an additive effect between metabolic syndrome and AF, since the latter also increases the risk of ESRD by 51% (22). AF itself may increase the risk of ESRD by multiple mechanisms, including renin-angiotensin-aldosterone system activation, volume retention, heart failure aggravation, renal artery thromboembolism, and decreased cardiac output and renal perfusion due to rapid/irregular ventricular rate (23). Furthermore, metabolic syndrome may further aggravate the risk of ESRD among patients with AF. Risk prediction for ESRD could be improved if it is individualized according to a patient's metabolic status.

In this study, we compared the effect of each metabolic syndrome component. We found that the impact on the risk of incident ESRD varied across the five metabolic syndrome components (Supplementary Figure S1). The results suggest possible differences in the risk of ESRD among patients with AF and metabolic syndrome, depending on the diagnostic criteria they meet. Therefore, modifiable risk factors for ESRD should be identified and individualized management of AF is necessary to prevent ESRD.

### 4.1. Limitations

Some limitations of this study need to be addressed. First, because this study was retrospective, a nationwide cohort is needed to ascertain the causal relationship between metabolic syndrome and incident ESRD. Second, the five metrics used for defining metabolic syndrome, especially blood pressure, may vary from time to time among patients with AF. Therefore, the reliability of the results may be a concern. Third, our results may not be applicable to the Western population because the definition of increased waist circumference was based on the Korean guideline (12). Fourth, although we observed consistent results across different multivariate Cox regression analyses, hidden confounders might have significantly affected the results. Fifth, the etiology of incident ESRD among patients is unknown in our study. We presume that most causes were hypertension or diabetic nephropathy, as they are the two major risk factors for ESRD. Sixth, there could be the potential influence of warfarin use on our results, considering its known impact on vascular calcification and renal function decline. According to Table 2, the proportion of warfarin use increased from 10.3% in MS_0_ to 24.3% in MS_5_. If the increased use of warfarin had a significant biasing effect on our results, we would expect to observe divergent outcomes between Model 4 and Model 3, because Model 4 incorporated the covariates from Model 3, along with the inclusion of oral anticoagulants and antiplatelet agents. However, Supplementary Table S4 demonstrates that both models yield comparable results. Based on these findings, we concluded that the potential bias arising from the increased use of warfarin might not be significant in our analysis. Seventh, this study could not analyze temporal trends in the associations between ESRD risks and metabolic scores. Metabolic scores could be dynamic and vary as patients age or receive medical management. However, this study utilized cross-sectional health check-up data, and therefore the dataset did not contain serial health check-up data for the study population. A further study is warranted to investigate the impact of temporal changes in metabolic status on the risk of ESRD. Eighth, the difference in the classes of antihypertensive and antidiabetic medications across groups could be potential bias in our study. To further investigate this issue, we analyzed the use of different drug classes among the groups, as presented in Supplementary Table S5. Our analysis revealed that the most used antihypertensive drug class was angiotensin receptor blockers, while the least used drug class was angiotensin-converting enzyme inhibitors, with similar patterns observed across the groups (excluding MS_0_, where the use of antihypertensive drugs would not be expected). Furthermore, regardless of the metabolic score groups, the two most used antidiabetic drugs were metformin and sulfonylurea. Based on this analysis, it appears that the distribution of drug classes was similar among the different metabolic score groups. Ninth, although the use of OAC may prevent thromboembolic events, such as renal infarction, and potentially reduce the risk of incident ESRD, our study did not observe any significant interaction (Supplementary Table S3; *P*-for-interaction = .115). This lack of significant interaction could be attributed to the relatively low number of events among the subgroup with OAC use. Finally, this study has the potential for selection bias because it excluded patients who did not have health check-ups within two years of AF diagnosis. Furthermore, patients with longer AF durations may have results different from those of this study.

## 5. Conclusions

Metabolic syndrome is associated with an increased risk of incident ESRD in patients with AF. Metabolic syndrome components have an additive impact on the risk for incident ESRD. Among the five diagnostic criteria for metabolic syndrome, elevated blood pressure and impaired glycemic control were the most significant predictors, while increased waist circumference was not. Careful monitoring of declining renal function is advisable in patients with AF and severe metabolic syndrome.

## Data Availability

The raw data are available to researchers on relevant request and with approval by the Korean National Health Insurance Sharing Service.

## References

[1] Ding WY Gupta D Wong CF Lip GYH. Pathophysiology of atrial fibrillation and chronic kidney disease. Cardiovasc Res. (2021) 117(4):1046–59. 32871005 10.1093/cvr/cvaa258

[2] Watanabe H Watanabe T Sasaki S Nagai K Roden DM Aizawa Y. Close bidirectional relationship between chronic kidney disease and atrial fibrillation: the niigata preventive medicine study. Am Heart J. (2009) 158(4):629–36. 19781424 10.1016/j.ahj.2009.06.031

[3] Chen TH Chu YC Ou SM Tarng DC. Associations of atrial fibrillation with renal function decline in patients with chronic kidney disease. Heart. (2022) 108(6):438–44. 10.1136/heartjnl-2021-319297 34193464

[4] Hindricks G Potpara T Dagres N Arbelo E Bax JJ Blomstrom-Lundqvist C 2020 ESC guidelines for the diagnosis and management of atrial fibrillation developed in collaboration with the European association for cardio-thoracic surgery (EACTS): the task force for the diagnosis and management of atrial fibrillation of the European society of cardiology (ESC) developed with the special contribution of the European heart rhythm association (EHRA) of the ESC. Eur Heart J. (2021) 42(5):373–498. 10.1093/eurheartj/ehaa612 32860505

[5] Olesen JB Lip GY Kamper AL Hommel K Kober L Lane DA Stroke and bleeding in atrial fibrillation with chronic kidney disease. N Engl J Med. (2012) 367(7):625–35. 10.1056/NEJMoa1105594 22894575

[6] Romagnani P Remuzzi G Glassock R Levin A Jager KJ Tonelli M Chronic kidney disease. Nat Rev Dis Primers. (2017) 3:17088. 10.1038/nrdp.2017.88 29168475

[7] Lee SR Choi EK Han KD Cha MJ Oh S. Trends in the incidence and prevalence of atrial fibrillation and estimated thromboembolic risk using the CHA(2)DS(2)-VASc score in the entire Korean population. Int J Cardiol. (2017) 236:226–31. 10.1016/j.ijcard.2017.02.039 28233629

[8] Ahn HJ Lee H Park HE Han D Chang HJ Chun EJ Changes in metabolic syndrome burden and risk of coronary artery calcification progression in statin-naive young adults. Atherosclerosis. (2022) 360:27–33. 10.1016/j.atherosclerosis.2022.09.011 36257122

[9] Alberti KG Eckel RH Grundy SM Zimmet PZ Cleeman JI Donato KA Harmonizing the metabolic syndrome: a joint interim statement of the international diabetes federation task force on epidemiology and prevention; national heart, lung, and blood institute; American heart association; world heart federation; international atherosclerosis society; and international association for the study of obesity. Circulation. (2009) 120(16):1640–5. 10.1161/CIRCULATIONAHA.109.192644 19805654

[10] Kwon S Lee SR Choi EK Jung JH Han KD Oh S Hypertension control and end-stage renal disease in atrial fibrillation: a nationwide population-based cohort study. Clin Res Cardiol. (2022) 111(3):284–93. 10.1007/s00392-021-01899-8 34216251

[11] Choi EK. Cardiovascular research using the Korean national health information database. Korean Circ J. (2020) 50(9):754–72. 10.4070/kcj.2020.0171 32725984 PMC7441000

[12] Seo MH Lee WY Kim SS Kang JH Kang JH Kim KK 2018 Korean society for the study of obesity guideline for the management of obesity in Korea. J Obes Metab Syndr. (2019) 28(1):40–5. 10.7570/jomes.2019.28.1.40 31089578 PMC6484940

[13] van der Burgh AC Geurts S Ikram MA Hoorn EJ Kavousi M Chaker L. Bidirectional association between kidney function and atrial fibrillation: a population-based cohort study. J Am Heart Assoc. (2022) 11(10):e025303. 10.1161/JAHA.122.025303 35579615 PMC9238570

[14] Zebe H. Atrial fibrillation in dialysis patients. Nephrol Dial Transplant. (2000) 15(6):765–8. 10.1093/ndt/15.6.765 10831625

[15] Steffel J Collins R Antz M Cornu P Desteghe L Haeusler KG 2021 European heart rhythm association practical guide on the use of non-vitamin K antagonist oral anticoagulants in patients with atrial fibrillation. Europace. (2021) 23(10):1612–76. 10.1093/europace/euab065 33895845 PMC11636576

[16] Yang F Chou D Schweitzer P Hanon S. Warfarin in haemodialysis patients with atrial fibrillation: what benefit? Europace. (2010) 12(12):1666–72. 10.1093/europace/euq387 21045011

[17] ELIQUIS (apixaban). Product monograph: Brystol-Myers Squibb Co. (2011). 10.1093/cvr/cvaa258

[18] Pokorney SD Chertow GM Al-Khalidi HR Gallup D Dignacco P Mussina K Apixaban for patients with atrial fibrillation on hemodialysis: a multicenter randomized controlled trial. Circulation. (2022) 146(23):1735–45. 10.1161/CIRCULATIONAHA.121.054990 36335914

[19] Weldegiorgis M Woodward M. The impact of hypertension on chronic kidney disease and end-stage renal disease is greater in men than women: a systematic review and meta-analysis. BMC Nephrol. (2020) 21(1):506. 10.1186/s12882-020-02151-7 33238919 PMC7687699

[20] Shen Y Cai R Sun J Dong X Huang R Tian S Diabetes mellitus as a risk factor for incident chronic kidney disease and end-stage renal disease in women compared with men: a systematic review and meta-analysis. Endocrine. (2017) 55(1):66–76. 10.1007/s12020-016-1014-6 27477292

[21] Wu N Qin Y Chen S Yu C Xu Y Zhao J Association between metabolic syndrome and incident chronic kidney disease among Chinese: a nation-wide cohort study and updated meta-analysis. Diabetes Metab Res Rev. (2021) 37(7):e3437. 10.1002/dmrr.3437 33469988

[22] O'Neal WT Tanner RM Efird JT Baber U Alonso A Howard VJ Atrial fibrillation and incident end-stage renal disease: the REasons for geographic and racial differences in stroke (REGARDS) study. Int J Cardiol. (2015) 185:219–23. 10.1016/j.ijcard.2015.03.104 25797681 PMC4621209

[23] McManus DD Saczynski JS Ward JA Jaggi K Bourrell P Darling C The relationship between atrial fibrillation and chronic kidney disease: epidemiologic and pathophysiologic considerations for a dual epidemic. J Atr Fibrillation. (2012) 5(1):442. 28496745 10.4022/jafib.442 PMC5153080

