# Supplementary material for: Impact of components of metabolic syndrome on the risk of adverse renal outcomes in patients with atrial fibrillation: a nationwide cohort study
Source: Front Cardiovasc Med. 2023 Oct 17;10:1208979. doi: 10.3389/fcvm.2023.1208979 (PMC10616466; doi:10.3389/fcvm.2023.1208979)
Supplement: Supplementary file 1 [file Datasheet1.docx]

Supplementary Material

Impact of Components of Metabolic Syndrome on the Risk of Adverse Renal Outcomes in Patients With Atrial fibrillation: A Nationwide Cohort Study

Soonil Kwon, MD,^1,†^ So-Ryoung Lee, MD, PhD,^1,†^ Eue-Keun Choi, MD, PhD,^1,2,*^ Seung-Woo Lee, MS,^3^ Jin-Hyung Jung, MS,^3^ Kyung-Do Han, PhD,^4^ Hyo-Jeong Ahn, MD,^1^ Seil Oh, MD, PhD, FHRS,^1,2^ Gregory Y. H. Lip, MD^1,2,5,6^

^1^Department of Internal Medicine, Seoul National University Hospital, Seoul, Republic of Korea

^2^Department of Internal Medicine, Seoul National University College of Medicine, Seoul, Republic of Korea

^3^Department of Medical Statistics, College of Medicine, The Catholic University of Korea, Seoul, Republic of Korea

^4^Department of Statistics and Actuarial Science, Soongsil University, Seoul, Republic of Korea

^5^Liverpool Centre for Cardiovascular Science at University of Liverpool, Liverpool John Moores University and Liverpool Chest & Heart Hospital, Liverpool, United Kingdom

^6^Department of Clinical Medicine, Aalborg University, Aalborg, Denmark

**† Joint first authors.**

*** Correspondence:**Eue-Keun Choi, MD, PhD

Department of Internal Medicine, Seoul National University College of Medicine and Seoul National University Hospital

101 Daehak-ro, Jongno-gu, Seoul, 03080, Republic of Korea

Phone: +82-2-2072-0688 / Fax: +82-2-762-9662

E-mail: [choiek17@snu.ac.kr](mailto:choiek17@snu.ac.kr)

# Supplementary Tables and Figures

## Supplementary Tables

**Supplementary Table 1.** Operational definitions of the study covariates

|  | ICD-10-CM code | Operational definition |
| --- | --- | --- |
| Inclusion/exclusion criteria |  |  |
| Atrial fibrillation | I48.0-48.4, I48.9 | Admission or outpatient department≥2 |
| End stage renal disease | N18.5 or Z49 | Dialysis ≥2 |
| Mitral stenosis or prosthetic heart valves | I05.0, I05.2, I05.9, Z95.2-Z95.4 | Admission or outpatient department≥1 |
| Health habits and low-income status^a^ |  |  |
| Smoking | None | Defined as a current smoker according to the response to the health survey provided by the NHIS. |
| Alcohol drinking | None | Defined as a current drinker according to the response to the health survey provided by the NHIS. |
| Regular exercise | None | Moderate physical activity (more than 30 minutes ≥5 days/week) or strenuous physical activity (more than 20 minutes ≥3 days/week), based on the responses to the health survey provided by the NHIS. |
| Low-income status | None | Defined as if a responder belonged to the lower 20 percentile of the answers of income with medical aid from the survey provided by the NHIS. |
| Comorbidities |  |  |
| Hypertension | I10-I13, I15; and minimum 1 prescription of anti-hypertensive drug (thiazide, loop diuretics, aldosterone antagonist, alpha-/beta-blocker, calcium-channel blocker, angiotensin-converting enzyme inhibitor, angiotensin II receptor blocker). | Admission≥1 or outpatient department≥2 |
| Diabetes mellitus | E11-E14; and minimum 1 prescription of anti-diabetic drugs (sulfonylureas, metformin, meglitinides, thiazolidinediones, dipeptidyl peptidase-4 inhibitors, α-glucosidase inhibitors and insulin). | Admission≥1 or outpatient department≥2 |
| Dyslipidemia | E78 | Admission or outpatient department≥1 |
| Ischemic heart disease | I21, I22 | Admission or outpatient department≥1 |
| Heart failure | I50 | Admission or outpatient department≥1 |
| Ischemic stroke | I63, I64 | Admission or outpatient department≥1 |
| Peripheral artery disease | I70, I73 | Admission or outpatient department≥2 |
| Chronic obstructive pulmonary disease | J41-44 | Admission≥1 |
| Any malignancy | C00-97 and RID code (V193) | Admission or outpatient department≥1 |
| Chronic kidney disease | None | eGFR <60 mL/min/1.73m² |

Demographic variables and comorbidities are measured at the health check-up after the diagnosis of AF.

^a^ The definition is based on the questionnaire used during the health check-ups provided by the NHIS.

Abbreviations: COPD, chronic obstructive pulmonary disease; ICD-10-CM, International Classification of Diseases, Tenth Revision, Clinical Modification; eGFR, estimated glomerular filtration rate calculated; MACE, major adverse cardiovascular event; NHIS, National Health Insurance Service; RID, Rare Intractable Disease.

**Supplementary Table 2.** A complete list of statistical models for multivariate Cox regression analyses

| Statistical models | Covariates for adjustment |
| --- | --- |
| Model 1 | None |
| Model 2 | Age and sex |
| Model 3 | Covariates in Model 2, body mass index, health habits (including alcohol drinking, smoking, and regular exercise), and low-income status |
| Model 4 | Covariates in Model 3, and concomitant drugs (including oral anticoagulants and antiplatelet agents) |
| Model 5 | Covariates in Model 4, and estimated glomerular filtration rates |
| Model 6 | Covariates in Model 4, and chronic kidney disease (defined by estimated glomerular filtration rate <60 ml/kg/1.73m^2^) |
| Model 7 | Covariates in Model 4, and chronic kidney disease (defined by Table S1) |
| Model 8 | Covariates in Model 5, and comorbidities (including ischemic heart disease, heart failure, stroke, peripheral artery disease, chronic obstructive pulmonary disease, and any malignancy) |
| Model 9 | Covariates in Model 6, and comorbidities (including ischemic heart disease, heart failure, stroke, peripheral artery disease, chronic obstructive pulmonary disease, and any malignancy) |
| Model 10 | Covariates in Model 7, and comorbidities (including ischemic heart disease, heart failure, stroke, peripheral artery disease, chronic obstructive pulmonary disease, and any malignancy) |
| Model 11 (the final model) | Covariates in Model 8, and the 5 metrics used in the definition of the metabolic syndrome (including waist circumference, fasting blood glucose, blood pressure, triglyceride, and high-density lipoprotein cholesterol) |

**Supplementary Table 3.** Subgroup analyses

| Subgroup | Metabolic score | N | ESRD | Crude incidence rate, 1000 PY | Adjusted HR (95% CI) | *P*-for-interaction |
| --- | --- | --- | --- | --- | --- | --- |
| CHA_2_DS_2_-VASc scores 0~1 | MS_0_ | 9889 | 5 | 0.12 | Reference | 0.966 |
|  | MS_1_ | 12,926 | 11 | 0.20 | 0.93 (0.31–2.79) |  |
|  | MS_2_ | 12,407 | 11 | 0.22 | 1.02 (0.32–3.24) |  |
|  | MS_3_ | 10,047 | 15 | 0.37 | 1.62 (0.49–5.33) |  |
|  | MS_4_ | 5897 | 9 | 0.39 | 1.71 (0.44–6.69) |  |
|  | MS_5_ | 1760 | 2 | 0.30 | 1.32 (0.19–8.96) |  |
| CHA_2_DS_2_-VASc scores ≥2 | MS_0_ | 2858 | 3 | 0.28 | Reference |  |
|  | MS_1_ | 18,133 | 44 | 0.64 | 1.29 (0.40–4.17) |  |
|  | MS_2_ | 27,954 | 96 | 0.93 | 1.61 (0.51–5.09) |  |
|  | MS_3_ | 38,021 | 186 | 1.35 | 2.10 (0.67–6.62) |  |
|  | MS_4_ | 40,733 | 298 | 2.12 | 2.53 (0.80–7.97) |  |
|  | MS_5_ | 21,809 | 195 | 2.68 | 3.20 (1.00–10.20) |  |
| Males | MS_0_ | 6808 | 5 | 0.18 | Reference | 0.838 |
|  | MS_1_ | 19,301 | 43 | 0.57 | 1.93 (0.76–4.89) |  |
|  | MS_2_ | 25,103 | 81 | 0.86 | 2.49 (1.00–6.20) |  |
|  | MS_3_ | 28,403 | 142 | 1.37 | 3.46 (1.40–8.58) |  |
|  | MS_4_ | 27,150 | 198 | 2.12 | 4.20 (1.69–10.45) |  |
|  | MS_5_ | 13,168 | 125 | 2.87 | 5.88 (2.31–14.97) |  |
| Females | MS_0_ | 5939 | 3 | 0.12 | Reference |  |
|  | MS_1_ | 11,758 | 12 | 0.25 | 1.44 (0.40–5.22) |  |
|  | MS_2_ | 15,258 | 26 | 0.44 | 1.84 (0.54–6.32) |  |
|  | MS_3_ | 19,665 | 59 | 0.80 | 2.91 (0.86–9.78) |  |
|  | MS_4_ | 19,480 | 109 | 1.55 | 3.78 (1.12–12.77) |  |
|  | MS_5_ | 10,401 | 72 | 2.01 | 4.31 (1.23–15.16) |  |
| eGFR ≥60 mL/kg/1.73m^2^ | MS_0_ | 12,147 | 7 | 0.14 | Reference | 0.010 |
|  | MS_1_ | 28,017 | 23 | 0.21 | 0.90 (0.38–2.12) |  |
|  | MS_2_ | 35,051 | 45 | 0.34 | 1.17 (0.52–2.67) |  |
|  | MS_3_ | 40,218 | 59 | 0.40 | 1.32 (0.58–3.02) |  |
|  | MS_4_ | 37,085 | 74 | 0.57 | 1.57 (0.68–3.63) |  |
|  | MS_5_ | 18,012 | 33 | 0.54 | 1.26 (0.50–3.15) |  |
| eGFR <60 mL/kg/1.73m^2^ | MS_0_ | 600 | 1 | 0.37 | Reference |  |
|  | MS_1_ | 3042 | 32 | 2.56 | 7.53 (1.03–55.33) |  |
|  | MS_2_ | 5310 | 62 | 3.01 | 9.27 (1.28–67.31) |  |
|  | MS_3_ | 7850 | 142 | 4.88 | 14.93 (2.07–107.83) |  |
|  | MS_4_ | 9545 | 233 | 7.03 | 19.33 (2.67–139.88) |  |
|  | MS_5_ | 5557 | 164 | 9.15 | 28.37 (3.88–207.47) |  |
| Without OAC use | MS_0_ | 11,217 | 7 | 0.15 | Reference | 0.115 |
|  | MS_1_ | 24,718 | 38 | 0.37 | 1.43 (0.69–2.96) |  |
|  | MS_2_ | 30,949 | 89 | 0.72 | 2.05 (1.02–4.12) |  |
|  | MS_3_ | 34,505 | 147 | 1.09 | 2.73 (1.37–5.47) |  |
|  | MS_4_ | 31,760 | 218 | 1.82 | 3.62 (1.80–7.28) |  |
|  | MS_5_ | 15,452 | 137 | 2.43 | 4.92 (2.37–10.18) |  |
| With OAC use | MS_0_ | 1530 | 1 | 0.18 | Reference |  |
|  | MS_1_ | 6341 | 17 | 0.90 | 2.35 (0.32–17.52) |  |
|  | MS_2_ | 9412 | 18 | 0.59 | 1.46 (0.20–11.00) |  |
|  | MS_3_ | 13563 | 54 | 1.28 | 2.37 (0.32–17.39) |  |
|  | MS_4_ | 14870 | 89 | 2.01 | 2.46 (0.34–18.10) |  |
|  | MS_5_ | 8117 | 60 | 2.61 | 3.31 (0.44–24.94) |  |

MS_0_~MS_5_ denotes the populations with a metabolic score of 0~5, accordingly. Abbreviations: CI, confidence interval; eGFR, estimated glomerular filtration rate; ESRD, end stage renal disease; HR, hazard ratio; OAC, oral anticoagulant; PY, person-year.

**Supplementary Table 4.** Sensitivity analyses - Comparison of the results with different statistical models

| Groups | Adjusted HR (95% CI),  Model 1 | Adjusted HR (95% CI),  Model 2 | Adjusted HR (95% CI),  Model 3 | Adjusted HR (95% CI),  Model 4 | Adjusted HR (95% CI),  Model 5 | Adjusted HR (95% CI),  Model 6 | Adjusted HR (95% CI),  Model 7 | Adjusted HR (95% CI),  Model 8 | Adjusted HR (95% CI),  Model 9 | Adjusted HR (95% CI),  Model 10 | Adjusted HR (95% CI),  Model 11  (the final model) |
| --- | --- | --- | --- | --- | --- | --- | --- | --- | --- | --- | --- |
| MS_0_ | Reference | Reference | Reference | Reference | Reference | Reference | Reference | Reference | Reference | Reference | Reference |
| MS_1_ | 2.87 (1.37–6.03) | 2.16 (1.03–4.54) | 2.30 (1.09–4.83) | 2.27 (1.08–4.78) | 2.11 (1.00–4.44) | 2.17 (1.03–4.58) | 2.20 (1.04–4.63) | 2.04 (0.97–4.30) | 2.12 (1.01–4.47) | 2.15 (1.02–4.53) | 1.65 (0.78–3.48) |
| MS_2_ | 4.47 (2.18–9.16) | 3.19 (1.55–6.54) | 3.58 (1.74–7.36) | 3.53 (1.71–7.26) | 3.09 (1.50–6.38) | 3.10 (1.50–6.39) | 3.25 (1.58–6.71) | 2.96 (1.43–6.10) | 2.99 (1.45–6.17) | 3.15 (1.53–6.51) | 2.08 (1.01–4.31) |
| MS_3_ | 7.29 (3.56–14.8) | 5.10 (2.51–10.35) | 5.85 (2.87–11.91) | 5.74 (2.81–11.69) | 4.55 (2.22–9.30) | 4.61 (2.25–9.41) | 4.84 (2.37–9.89) | 4.29 (2.10–8.78) | 4.37 (2.14–8.94) | 4.63 (2.26–9.47) | 2.94 (1.43–6.06) |
| MS_4_ | 12.1 (6.00–24.42) | 8.28 (4.10–16.73) | 9.74 (4.80–19.78) | 9.51 (4.67–19.35) | 6.78 (3.32–13.85) | 6.93 (3.40–14.15) | 7.28 (3.57–14.87) | 6.22 (3.04–12.73) | 6.43 (3.15–13.14) | 6.81 (3.33–13.92) | 3.71 (1.80–7.66) |
| MS_5_ | 16.08 (7.93–32.60) | 11.05 (5.44–22.44) | 14.39 (6.99–29.62) | 14.03 (6.80–28.94) | 9.86 (4.76–20.44) | 9.69 (4.68–20.05) | 9.61 (4.64–19.91) | 8.96 (4.32–18.58) | 8.88 (4.29–18.40) | 8.97 (4.32–18.60) | 4.82 (2.29–10.15) |

MS_0_~MS_5_ denotes the populations with a metabolic score of 0~5, accordingly. Model 1 used no covariate adjustment. Model 2 adjusted age and sex. Model 3 adjusted the covariates in Model 2, body mass index, health habits (including alcohol drinking, smoking, and regular exercise), and low-income status. Model 4 adjusted the covariates in Model 3, and concomitant drugs (including oral anticoagulants and antiplatelet agents). Model 5 adjusted the covariates in Model 4 and estimated glomerular filtration rates. Model 6 adjusted the covariates in Model 4, and chronic kidney disease (defined by estimated glomerular filtration rate <60 ml/kg/1.73m^2^). Model 7 adjusted the covariates in Model 4, and chronic kidney disease. Model 8 adjusted the covariates in Model 5, and comorbidities (including ischemic heart disease, heart failure, stroke, peripheral artery disease, chronic obstructive pulmonary disease, and any malignancy). Model 9 adjusted the covariates in Model 6, and comorbidities (including ischemic heart disease, heart failure, stroke, peripheral artery disease, chronic obstructive pulmonary disease, and any malignancy). Model 10 adjusted the covariates in Model 7, and comorbidities (including ischemic heart disease, heart failure, stroke, peripheral artery disease, chronic obstructive pulmonary disease, and any malignancy). Model 11 adjusted the covariates in Model 8, and the 5 metrics used in the definition of the metabolic syndrome (including waist circumference, fasting blood glucose, blood pressure, triglyceride, and high-density lipoprotein cholesterol). Abbreviations: CI, confidence interval; HR, hazard ratio; PY, person-year.

**Supplementary Table 5. Antihypertensive and antidiabetic drug uses among groups**

|  | MS_0_ (n=12,747) | MS_1_ (n=31,059) | MS_2_ (n=40,361) | MS_3_ (n=48,068) | MS_4_ (n=46,630) | MS_5_ (n=23,569) | *P* |
| --- | --- | --- | --- | --- | --- | --- | --- |
| Antidiabetic drugs |  |  |  |  |  |  |  |
| Sulfonylurea | 0 (0) | 496 (1.6) | 2381 (5.9) | 3898 (8.1) | 8701 (18.7) | 6995 (29.7) | <.0001 |
| Meglitinide | 0 (0) | 31 (0.1) | 137 (0.3) | 201 (0.4) | 484 (1.0) | 329 (1.4) | <.0001 |
| Metformin | 0 (0) | 795 (2.6) | 3567 (8.8) | 5782 (12.0) | 13,163 (28.2) | 10,379 (44.0) | <.0001 |
| Thiazolidinedione | 0 (0) | 62 (0.2) | 339 (0.8) | 605 (1.3) | 1408 (3.0) | 1336 (5.7) | <.0001 |
| Alpha-glucosidase inhibitors | 0 (0) | 111 (0.4) | 544 (1.4) | 855 (1.8) | 1799 (3.9) | 1218 (5.2) | <.0001 |
| Dipeptidyl peptidase-4 inhibitors | 0 (0) | 350 (1.1) | 1553 (3.9) | 2739 (5.7) | 7069 (15.2) | 5889 (25.0) | <.0001 |
| Insulin | 0 (0) | 528 (1.7) | 1715 (4.3) | 2437 (5.1) | 5320 (11.4) | 4070 (17.3) | <.0001 |
| Antihypertensive drugs |  |  |  |  |  |  |  |
| Beta blockers | 0 (0) | 6370 (20.5) | 10,025 (24.8) | 14,958 (31.1) | 17,215 (36.9) | 9613 (40.8) | <.0001 |
| Calcium channel blockers | 0 (0) | 6037 (19.4) | 10,970(27.2) | 16,333 (34.0) | 19,426 (41.7) | 11,786 (50.0) | <.0001 |
| Angiotensin-converting enzyme inhibitors | 0 (0) | 1416 (4.6) | 2200 (5.5) | 3495 (7.3) | 4169 (8.9) | 2128 (9.0) | <.0001 |
| Angiotensin receptor blockers | 0 (0) | 7538 (24.3) | 13,481 (33.4) | 20,948 (43.6) | 25,679 (55.1) | 15,581 (66.1) | <.0001 |
| Diuretics | 0(0) | 2438(7.85) | 4859(12.04) | 7431(15.46) | 9503(20.38) | 5951(25.25) | <.0001 |

## Supplementary Figures

**Supplementary Figure 1.** Impact of each diagnostic criterion of metabolic syndrome on incident ESRD


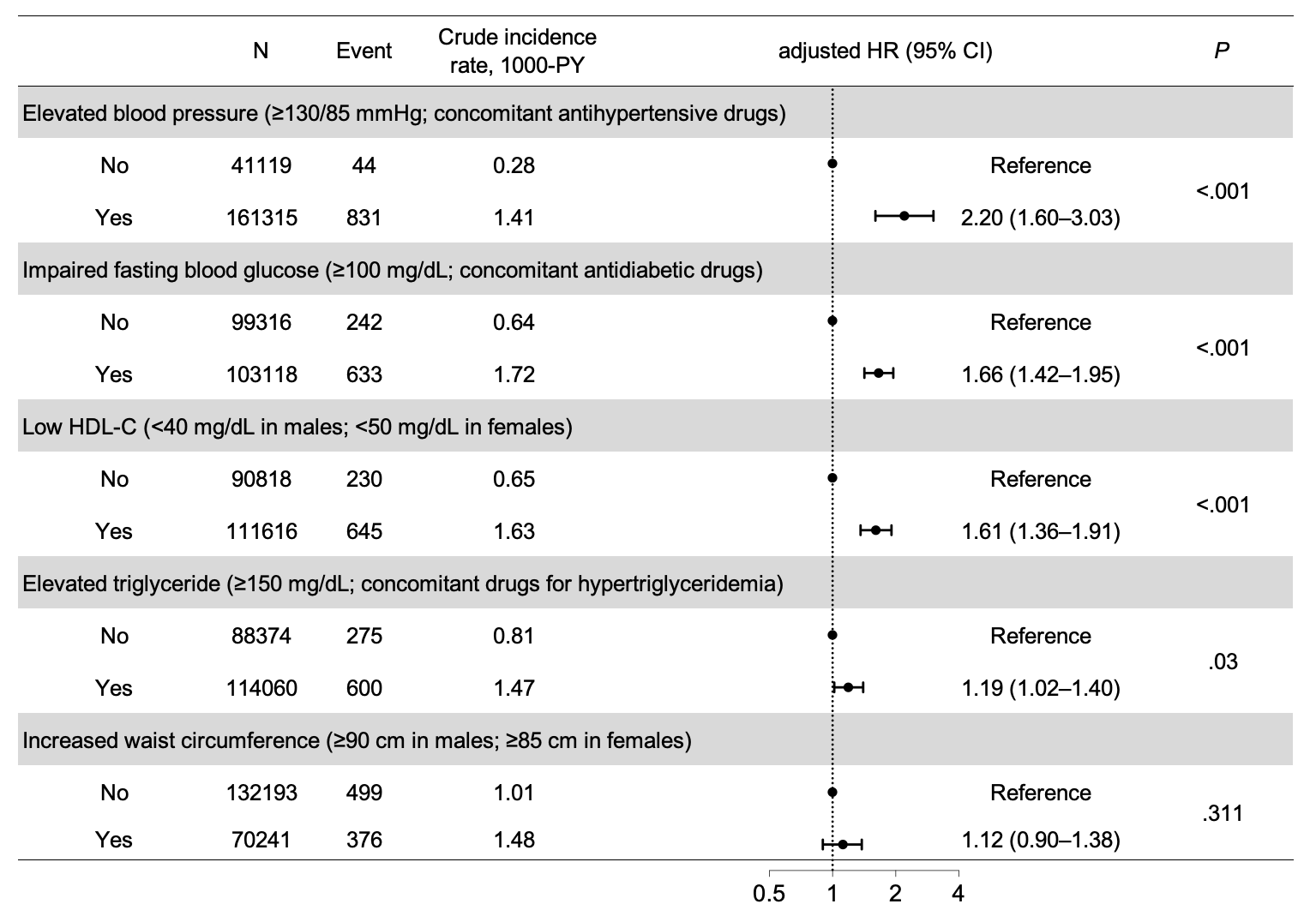


Except for increased waist circumference, the other 4 diagnostic criteria of metabolic syndrome were associated with increased risks of incident ESRD among AF patients. Abbreviations: AF, atrial fibrillation; CI, confidence interval; ESRD, end-stage renal disease; HR, hazard ratio; PY, person-year.
